# Monocyte recruitment in venous pulmonary embolism at time of cancer diagnosis in upper gastrointestinal cancer patients

**DOI:** 10.1007/s11239-023-02897-5

**Published:** 2023-10-04

**Authors:** Sarah S. Jakobsen, Jens B. Frøkjaer, Rune V. Fisker, Søren R. Kristensen, Ole Thorlacius-Ussing, Anders C. Larsen

**Affiliations:** 1 Department of Gastrointestinal Surgery, Aalborg University Hospital, Hobrovej 18-22, 9000 Aalborg, Denmark; 2 Department of Radiology, Aalborg University Hospital, 9000 Aalborg, Denmark; 3 Department of Biochemistry, Aalborg University Hospital, 9000 Aalborg, Denmark; 4 Department of Nuclear Medicine, Aalborg University Hospital, 9000 Aalborg, Denmark; 5 Clinical Cancer Research Center, Aalborg University Hospital, 9000 Aalborg, Denmark; 6 Department of Clinical Medicine, Aalborg University, 9000 Aalborg, Denmark; 7 Cardiovascular Research Center, Aalborg University, 9000 Aalborg, Denmark

**Keywords:** Cancer, D-dimer, Monocytes, Pulmonary embolism, Venous thrombosis

## Abstract

Upper gastrointestinal cancer is frequently complicated by venous thromboembolisms (VTE), especially pulmonary embolisms (PE) increase the mortality rate. Monocytes are a part of the innate immune system and up-regulation may indicate an ongoing inflammatory response or infectious disease and has lately been associated with a moderate risk of suffering from VTE. This prospectively study aims to compare the incidence of pulmonary embolism with markers of coagulation and compare it to the absolute monocyte count. A consecutive cohort of 250 patients with biopsy proven upper gastrointestinal cancer (i.e. pancreas, biliary tract, esophagus and gastric cancer) where included at the time of cancer diagnosis and before treatment. All patients underwent bilateral compression ultrasonography for detection of deep vein thrombosis (DVT). Of these 143 had an additionally pulmonary angiografi (CTPA) with the staging computer tomography. 13 of 250 patients (5.2%) had a DVT and 11 of 143 (7.7%) had CTPA proven PE. PE was significantly more common among patients with elevated D-dimer (OR 11.62, 95%CI: 1.13–119, *P* = 0.039) and elevated absolute monocyte count (OR 7.59, 95%CI: 1.37–41.98, *P* = 0.020). Only patients with pancreatic cancer had a significantly higher risk of DVT (OR 11.03, 95%CI: 1.25–97.43, *P =* 0.031). The sensitivity of absolute monocyte count was 63.6 (95%CI: 30.8–89.1) and specificity 80.3 (95%CI: 72.5–86.7), with a negative predictive value of 96.4 (95%CI: 91–99) in PE. An increased absolute monocyte count was detected in patients suffering from PE but not DVT, suggesting a possible interaction with the innate immune system.

## Highlights

- Cancer patients have a greater risk of developing thromboembolisms, which could potentially have a fatal outcome, especially when developed in the lungs.
- In emergency rooms biomarkers in blood sample are used as a screening tool in detecting thromboembolisms, but do not differentiate between different types.
- Pulmonary embolisms may be asymptomatic. We included 250 upper gastrointestinal cancer patients and screened for thromboembolisms in the legs and lungs.
- We tested monocytes as a biomarker to detect Pulmonary embolisms. Total monocyte count increased in case of lunge embolisms but not when a thrombosis was solely in the legs.
- Thereby monocytes may be used as a screening tool when suspecting lung embolisms, though patients may not have relevant symptoms.
- The detection of pulmonary embolisms is important as it effects the mortality rate. It could help determine who should receive anticoagulant treatment at an earlier point.

## Introduction

A common complication of cancer is risk of developing venous thromboembolisms (VTE) and the mortality rate is higher among patients developing pulmonary embolism (PE) compared to deep vein thrombosis (DVT) [1].

Initial clinical suspicion of PE derives from symptoms such as shortness of breath, chest pain and dizziness [2, 3]. Though, PE can be without symptoms and only detected incidental on computer tomography (CT) performed on other indications [4]. Autopsies of patients have revealed PE as a common cause of death [5], and mortality may be independent of whether VTE is symptomatic or found incidentally [6]. PE is still only recognized with either pulmonary lung scintigraphy or computer tomography pulmonary angiography [7].

D-dimer is a product of fibrin degradation and has use as a negative predictive value in the primary screening of patients suspected of VTE. If the d-dimer is not increased, the risk of VTE occurrence is low but can be enhanced by infection, cancer, different medical treatments, and various other medical disorders. D-dimer does not differentiate between PE and DVT, and no single blood test uniquely identifies venous pulmonary embolisms [8].

Monocytes can differentiate into macrophages and dendrite cells which act as antigen-presenting cells in the host defense against pathogens. An upregulation of the absolute monocyte count in the peripheral blood may indicate an ongoing inflammatory response or an infectious disease [9].

Monocytes are the major contributor of tissue factor (TF) and are an important part of the blood thrombogenicity. In the Tromsø Study a survey of 25.127 subjects, 429 incidents of VTE events were registered in the discharge diagnosis registry. Subjects with a monocyte count higher than 0.7 10E9/L had a hazard ratio of 2.5 (95%CI 0.69–9.12) of suffering from a VTE within the first year in comparison to subjects with a normal monocyte count [10].

### Aim

This clinical prospective study aims to investigate the relation between absolute monocyte count and PE in untreated upper gastrointestinal cancer patients at time of cancer diagnosis, and compare it to known markers of coagulation: D-dimer, Thrombin-antithrombin complex (TAT) and Prothrombin Fragment 1 and 2 (F1 + 2).

## Methods

### Patients

Patients at Aalborg University Hospital with a biopsy proven or tentative diagnosis of upper Gastrointestinal cancer between February 2008 and February 2011, was included in this cross-sectional study as previously described [11]. Eligible patients were consecutively included after written and oral informed consent had been obtained (Clinical Trials.gov: NCT00660205 Approval of local ethics committee of Region North Jutland, Denmark: N-20080002).

The information collected included age, gender, tumor location, and stage (according to Union for International Cancer Control 6. Edition of the TNM-classification) [12]. Data were collected using Epidata® software (The Epidata Association, Odense, Denmark) [13].

### Diagnosis and staging of cancer and VTE at inclusion

At time of inclusion patients had a biopsy to confirm cancer diagnosis. Before any medical treatment or operation, patients underwent diagnostic computer tomography (CT) of the thorax and abdomen or positron emission tomography CT (PET-CT). In addition, the patients were staged, using the TNM-classification system to group the patients according to UICC stages I–IV.

Bilateral compression ultrasonography (biCUS) includes the femoral, popliteal and calf veins and was performed according to standard procedures (grey scale B-mode with colour Doppler) using a high-end scanner (Esaote MyLab 70 XVG with LA332 11–3 MHz probe, Genoa, Italy). One of two experienced sonographers (> 10 years) performed all examinations.

From February 2009 CT or PET-CT scans used in the first routine staging of the cancer were modified and included an arterial-phase scan covering the pulmonary arteries to diagnose PE, a so-called CT pulmonary angiogram (CTPA). The CTPA was done as a state-of-the-art examination.

### Blood samples

Blood samples were collected on admission before the start of diagnostic work up and treatment. Blood samples were drawn by venipuncture according to The European Concerted Action on Thrombosis (ECAT) procedures. Samples for plasma D-dimer were immediately analyzed as in-house routine analyses by the Auto Dimer assay (Biopool International, Umeaa, Sweden) as described by the manufacturer.

Plasma F1 + 2 and TAT were measured by commercially available enzyme-linked immunosorbent assay (ELISA) kits, as described by the manufacturer [Enzygnost F1þ2 (Dade Behring Marburg GmbH, Marburg, Germany), and Enzygnost TAT (Dade Behring Marburg GmbH), respectively]. The determinants for F1 + 2 and TAT are given as mean values of duplicate measurements.

The machine used in differentiating blood cell count was a ADCIA 2120I, at Aalborg University Hospital.

### Statistics

Plasma levels of F1 + 2 and TAT followed a normal distribution after logarithmic transformation. According to manufactor’s information for F1 + 2 and TAT, the median plasma level of F1 + 2 was 115 pmol/L (5–95% confidence interval 69–229 pmol/L). The mean plasma reference level for TAT was < 2.0 µg/L (2.5–97.2% percentile: < 2.0–4.2 µg/L). Upper limit of reference interval was used to discriminate between normal levels of F1 + 2 and TAT and increased levels. Due to left censoring at lower limit for the immunoassay analysis, D-dimer was interval censored at the cut-off level (< 0.3 mg/L) for the assay. This cut-off level was used to differentiate between normal and increased levels of D-dimer. Plasma levels of D-dimer did not follow a normal distribution. The monocyte count cut-off value of 0.7 × 10E9/L (in house reference level) and platelet count 350 × 10E9/L (from Khorana score) was determined based on inhouse analyses. Sensitivity, specificity, positive and negative predictive value were estimated using a r × c contingency table. The significance level was set to p < 0.05. A non-parametric receiver operating characteristic (ROC) plot was constructed. The results were expressed as negative predictive values (NPV), positive predictive values (PPV), specificity and sensitivity.

## Results

### Patients

From February 2008 to February 2011, 514 patients were deemed eligible for inclusion. Of these, 250 patients were followed and examined according to protocol, including screening for DVT. Routine CTPA was added to the protocol during the study and subsequently 143 patients were examined for the presence of PE and DVT. This addition created two different subgroups of patients; 250 patients screened for DVT, and, of these, 143 patients screened for DVT and PE. Baseline characteristics are shown in Table 1.

**Table 1:** Descriptive data

	DVT (+) (n = 250)			PE (+) (n = 143)		
	n (%)		p*	n (%)		p*
Sex						
Male	7/169 (4.1)			5/97 (5.2)		
Female	6/81 (7.4)		0.213	6/46 (13)		0.096
Age						
< 50	0/13 (0)			1/9 (11.1)		
50–70	5/167 (3)			3/94 (3.2)		
> 70	8/70 (11.4)		0.146	7/40 (17.5)		0.040
Tumor location						
Pancreas	9/95 (9.5)			7/50 (14)		
Biliary tract	2/22 (9.1)			2/13 (15.4)		
Gallbladder	0/4 (0)			0/2 (0)		
Lower esophagus	0/43 (0)			0/30 (0)		
Gastroesophageal junction	1/58 (1.7)			1/33 (3)		
Stomach	1/27 (3.7)			1/15 (6.7)		
Duodenum	0/1 (0)		0.152	0/0 (0)		0.126
Stage (UICC 6th)						
I	0/27 (0)			0/10 (0)		
II	1/71 (1.4)			0/40 (0)		
III	0/47 (0)			0/31 (0)		
IV	12/105 (11.4)		0.004	11/62 (17.7)		0.001
WHO Stage						
0	5/166 (3)			4/100 (4)		
1	5/57 (8.8)			5/33 (15.2)		
≥ 2	3/27 (11.1)		0.024	2/10 (20)		0.018
D-dimer						
< 0.3	0/131 (0)			0/80 (0)		
0.3–1	1/77 (1.3)			2/45 (4.4)		
> 1	12/42 (28.6)		0.000	9/18 (50)		0.000
Monocytes						
< 0.7	7/174 (4)			4/108 (3.7)		
0.7–2.0	5/61 (8.2)			7/35 (20)		
> 2.0	0/0 (0)		0.433**	0/0 (0)		0.177
Unknown	1/15 (6.7)			0/0 (0)		
Platelets						
≤ 350	8/152 (5.3)			7/94 (7.4)		
> 350	4/85 (4.71)			4/47 (8.2)		
Unknown	1/13 (7.69)		0.558*	0/2 (0.0)		0.531*
F1 + 2						
< 250	1/146 (0.7)			2/87 (2.3)		
250–750	7/90 (7.8)			4/49 (8.2)		
> 750	4/8 (50)		0.434**	5/6 (83.3)		0.461**
Unknown	1/6 (16.7)			0/1 (0)		
TAT						
< 1	1/112 (0.9)			1/63 (1.6)		
1–3	0/73 (0)			1/45 (2.2)		
> 3	11/59 (18)			9/34 (26.5)		
Unknown	1/6 (16.7)		0.110**	0/1 (0)		0.364**

Characteristics of the two subgroups: cancer-patients only developing a deep vein thrombosis (DVT), and cancer patients developing a pulmonary embolism (PE). Characteristics of gender, age, location and staging of tumor, world health organization (WHO) performance score, d-dimer, monocytes, prothrombin fragment 1 + 2 and thrombin-antithrombin complex (TAT). Significant when* p* < 0.05

*1-sided Fisher

**Chi^2

At the time of inclusion, the mean age of the 250 patients was 65 years (range 32–85) and 169 (67.6%) were men. The mean age of the subgroup of 143 patients was 64 years (range 32–80) and consisted of 97 (67.8%) men. Table 2 shows the distribution of DVT and PE among the 143 examined for both DVT and PE.

**Table 2:** Occurrence of venous thromboembolisms

		Pulmonary embolism		
		PE	None	Total
Deep vein thrombosis	DVT	8	2	10
	None	3	130	133
	Total	11	132	143

Description of the division of patients developing deep vein thrombosis (DVT), pulmonary embolism (PE), developing both or developing none of the thromboembolic states in 143 cancer patients

### Deep vein thrombosis

Table 1 shows the descriptive characteristics of the patients diagnosed with DVT and their blood values of D-dimer, monocytes, F1 + 2 and TAT at time of inclusion. In total 13 of the 250 patients developed DVT during the time of the study. Gender and age were not found significantly different, but all 13 of the patients had an age of 50 or more years. Of the 13 patients, 9 had a pancreatic primary tumor, and 12 of 13 had a cancer stage IV. Patients with DVT had a significantly elevated D-dimer (*P* = 0.000) with a cut-off value at 0.3 and all, except one patient, had a highly elevated D-dimer above 1 at time of inclusion. None of the other markers were significantly raised.

An univariable and multivariable regression analysis of the 250 patients are shown in Table 3, thereby determining risk factors of DVT at time of cancer diagnosis. In the univariable logistic regression tumor location, stage IV, WHO status performance above 0, elevated D-dimer above 0.3, elevated F1 + 2 above 250 and elevated TAT above 1 were determined as risk factors. In the multivariable logistic regression, tumor staging was the only risk factor of DVT.

**Table 3:** Regression analysis

	Univariable analysis			Multivariable analysis			
	Odds ratio (95%CI)		*p*	Odds ratio (95%CI)		*p*	
Deep vein thrombosis (*n* = 250)							
Sex	1.85 (0.60–5.70)		0.283				
Age	1.53 (0.49–4.83)		0.464				
PBC	6.35 (1.38–29.27)		0.018	3.81 (0.71–10.44)		0.118	
Stage IV	18.58 (2.38–145.29)		0.005	11.03 (1.25–97.43)		0.031	
WHO status	3.39 (1.07–10.71)		0.038	1.18 (0.30–4.61)		0.806	
D-dimer	21.86 (2.79–171.03)		0.003	2.89 (0.24–35.00)		0.404	
Monocytes	2.13 (0.69–6.57)		0.188				
Platelets	0.97 (0.31–3.05)		0.955				
F1 + 2	18.91 (2.42–147.90)		0.005	8.41 (0.90–78.56)		0.062	
TAT	10.57 (1.35–82.60)		0.025	1.97 (0.18–21.79)		0.579	
Pulmonary embolism (*n* = 143)							
Sex	2.76 (0.80–9.57)		0.110				
Age	1.97 (0.55–7.07)		0.295				
PBC	6.11 (1.27–29.37)		0.024	2.42 (0.40–14.68)		0.336	
Stage IV	1 NA		NA				
WHO status	4.67 (1.29–16.90)		0.019	2.39 (0.43–13.20)		0.317	
D-dimer	23 (2.85–185.74)		0.003	11.61 (1.13–119.17)		0.039	
Monocytes	7.13 (1.94–26.21)		0.003	7.59 (1.37–41.98)		0.020	
Platelets	1.10 (0.31–3.97)		0.879				
F1 + 2	8.14 (1.69–39.24)		0.009	4.82 (0.58–39.84)		0.144	
TAT	8.86 (1.10–71.17)		0.040	2.73 (0.24–30.91)		0.418	

A univariable regression and multivariable regression analysis to determine which values had a significant impact on the development of deep vein thrombosis or pulmonary embolism. The multivariable regression analysis was only performed on the significant (< 0.05) univariable values

* PBC* pancreaticobiliary cancer,* stage IV* IUCC stage 6. Edition,* WHO status* WHO performance status,* F1 + 2* fragment 1 + 2,* TTA* thrombin-antithrombin complex

### Pulmonary embolism

Among the 143 patients screened for DVT and PE, 11 patients developed PE. Men and women were represented equally, but a significant difference between age groups (*P* = 0.040) was found, as patients above 50 years had a higher tendency of developing PE. Of the 11 patients, 8 developed PE as well as DVT, as shown in Table 2. The characteristics of the 11 patients developing PE are shown in Table 1. Though no significant difference between site of primary cancer was found, 7 of 11 patients developing PE had a pancreatic cancer. All 11 patients had a cancer at stage IV, and 9 of 11 patients had a d-dimer above 1 at time of inclusion.

Determining the risk factors in Table 3, the univariable logistic regression showed tumor location, WHO performance status, d-dimer above 0.3, monocyte count above 0.7, F1 + 2 above 250 and TAT above 1 to be significant. When adjusting the multivariate logistic regression, d-dimer and monocyte count was significant risk factors (*P* = 0.039 and *P* = 0.020).

### Diagnostic accuracy

**Fig. 1:**
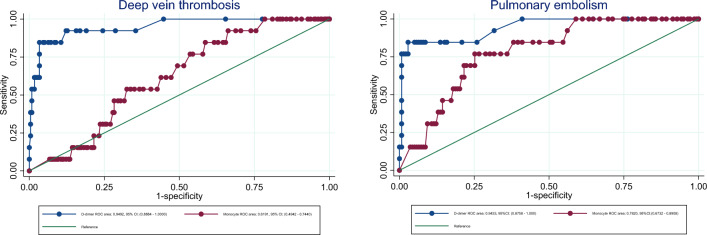
Specificity and sensitivity. Receiver operating characteristics (ROC) curve displaying specificity and sensitivity of D-dimer and monocyte count in prediction of deep vein thrombosis and pulmonary embolism

**Fig. 2:**
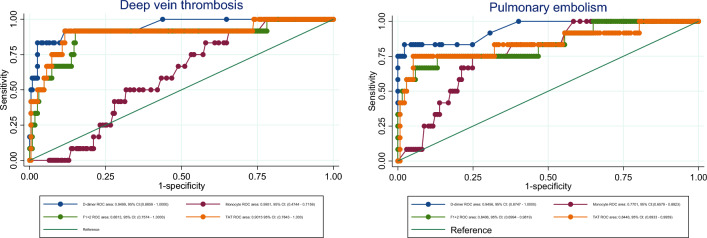
ROC curves: Monocyte count, d-dimer, F1 + 2 and TAT. ROC curves visualizing the specificity and sensitivity of monocyte count, d-dimer, F1 + 2 and TAT in case of DVT and pulmonary embolism

Figure 1 shows receiver operating characteristics (ROC) plot of D-dimer and monocytes in respectively DVT and PE. D-dimer had a high ROC area in both DVT and PE, whereas monocyte count was higher in PE (0.77) compared to DVT (0.62). An area < 0.75 is typically not clinically useful. Figure 2 compare the ROC curves of monocyte count, d-dimer, F1 + 2 and TAT in case of DVT and PE. The difference between DVT and PE in d-dimer, F1 + 2 and TAT was only ~ 0.01, whereas the difference between the DVT and PE monocyte count was ~ 0.15.

Table 4 lists the predictive values of negative predictive value, positive predictive value, specificity and sensitivity when using D-dimer and monocyte count in DVT and PE prediction.

**Table 4:** Predictive values

	NPV (%) (95%CI)	PPV (%) (95%CI)	Sensitivity (%) (95%CI)	Specificity (%) (95%CI)
DVT				
D-dimer	98.9 (94.2–100)	18 (8.6–31.4)	90 (55.5–99.7)	69.2 (60.6–76.9)
Monocyte	98.9 (94.2–100)	20 (10-33.7)	90.9 (58.7–99.8)	69.7 (61.1–77.4)
PE				
D-dimer	95.5 (89.7–98.5)	15.2 (5.1–31.9)	78.9 (71-85.5)	50 (18.7–81.3)
Monocyte	96.4 (91–99)	21.2 (9-38.9)	63.6 (30.8–89.1)	80.3 (72.5–86.7)

The predictive values shown as negative predictive value (NPV), positive predictive value (PPV), sensitivity and specificity of the use of D-dimer and monocyte count in the prediction of deep vein thrombosis (DVT) or pulmonary embolism (PE)

## Discussion

This prospective study found an association between increased absolute monocyte count in the cancer patients diagnosed with PE compared to DVT, suggesting that the embolus formation in PE may differ from the thrombus formation in DVT. To our knowledge this is the first time a blood test has shown significant difference between these two types of VTE.

A newly published retrospective study found a difference in blood sample parameters such as D-dimer, GFR, INR, creatinin and urea were significantly altered in case of PE, using machine learning algorithmics suggesting that this kind of information could elect candidates for CTPA [13]. In a substudy of the Cassini trial a heterogouns group of 124 ambulatory cancer patients was examined for various hemostatic factors and inflammatory biomarkers associated with a future risk of VTE [14]. Thus, further implementation of parameters, such as monocyte count, may improve the pre-test probability.

In the clinic, d-dimer is a highly valued screening tool, as it detects the amount of fibrin in the circulation. Fibrin has a high negative predictive value; however, elevation is less specific. All patients in our study, with a confirmed VTE, had a d-dimer > 0.3, confirming the negative predictive value [15].

But D-dimer does not distinguish among DVT and PE. In our study in contrast to the monocyte count which was significantly altered in case of PE but not in DVT, which prompt for further investigation of the immune system in VTE patients.

The Khorana score was developed for this measure. It utilizes five predictive variables: site of cancer, platelet count, hemoglobin, leukocyte count and body mass index. Depending on the score, a low, intermediate, or high risk of developing VTE is determined [16]. Blom et al. found tumors of the bone, ovary, brain, and pancreas at the highest risk of VTE [17]. Our study is partially in agreement. The location of the tumor was determined to be a significant predictor of both DVT and PE. Though our study focused on cancers of the gastrointestinal system, the highest occurrence of DVT were in pancreas cancer patients representing 69%, and 64% of the PE. Platelet count was not found significantly altered in either DVT or PE, thereby questioning the usability of this marker in the use of the Khorana score as previously reported by our study group [18]. Khorana score is designed to a mix of ambulatory cancer out-patients but seems less likely to identify VTE high-risk patients as reported in ovarian cancer and lung cancer [19, 20], and as we have previously shown that gastroesophageal cancer and gastric cancer may have a low frequency of VTE at the time of diagnosis, but the risk seems to increase upon start of cancer treatment, especially perioperative chemotherapy [21]. This could explain the difference in frequency of VTE and the various results. The small number of patients is a limitation of the present study but must be weighed against the unique screening with biCUS and CTPA at time of cancer diagnosis, which strengthen the VTE diagnosis.

In a recent retrospective study of 674 older patients with hip fracture Wang et al. found that a monocyte count above 0.6 × 10E9/L in a multivariate analysis was independently associated with a preoperative risk of DVT (OR 1.705 × 95%CI 1.12–2.59), but not for PE [22]. A register study with Mendelian Randomization on a genome-wide association study He et al. found that a genetically predicted monocyte count was negatively correlated with VTE, but does not discriminate between DVT and PE, and has no information about time of VTE examination blood sample drawn [23], and level of monocyte count. This is in accordance with Rezende et al. who found a low monocyte count (< 0.12 × 10E9/L) was invers related to risk of VTE, but with increasing risk of VTE (OR 2.75; 95%CI 1.3–5.8) at elevated monocyte count (> 0.77 × 10E9/L). The risk was higher in patients with PE alone (OR 1.53, 95%CI 0.77–3.04) than among patients with PE and DVT (OR 0.81, 95%CI 0.43–1.53) [24]. Together these studies support our results of a difference in absolute monocyte count and the development of PE.

Veins of the extremities differ macroscopically from the pulmonary arteries by the valves. The endothelium of the veins is normally intact and protect against coagulation but upon activation by either stasis or for instance inflammatory stimuli, this antithrombotic state can be harmed. Monocyte activation may release more tissue factor and stimulate the coagulation cascade resulting in thrombosis [25].

A hypoxic gradient as a result of pulmonary hypertension (PH), is a known complication to PE [26]. In our study we screened for PE at time of cancer diagnosis, though we had no way of determining the point of time of the PE occurrence. The elevated monocyte count could be a consequence of the altered lung structures. Florentin et al. showed monocytes recruitment to the perivascular spaces in the murine lung parenchyma and a subsequently macrophage differentiation as a response to affected tissue [27]. This is supported by other murine studies of hypoxia induced monocyte recruitment [28].

Kimball et al. showed that monocyte macrophage depletion had no effect on thrombogenesis but seemed to be involved in murine thrombus resolution [29]. This is in contrast with von Bruhl et al. who showed that patrolling monocytes on the endothelium may initiate DVT [30]. Both could be true but are more likely model dependent. Hanna et al. showed that murine patrolling monocytes seems to protect against lung metastasis in a lung cancer model and therefore could be activated in the present study leading to the elevated level of monocytes [31]. In another experimental model humane monocytes seem to influence size and density of the clot, and thereby the ability to migrate [32]. In conclusion monocytes like platelets and other haematopoic cells seem to be an integral part of the venous thrombogenesis [33, 34].

In conclusion, the absolute monocyte count was significantly increased in patients with pulmonary embolisms, and may indicate a possible interaction with the immune system. Further studies are needed to fully understand the usability of monocyte count in the diagnostic work up of VTE.

The authors wish to thank all he patients who participated in the study. We also thank the laboratory technicians J. Lundtoft and A. Nord, laboratory technicians at the Department of Gastroenterological Surgery, Aalborg University Hospital, for obtaining blood samples and providing logistic assistance, and nurse A. Østergaard Madsen and secretary A. Bahnsen for logistic assistance and patient administration. We also thank the following private foundations for supporting this study unconditionally: Karen Elise Jensen Foundation, Toyota Denmark Foundation, Arvid Nielson Foundation, Ejner Willumsen Foundation, and Civil Engineer Bent Bøgh and Wife Foundation. The authors also thank the private foundation Blegdalens Erhvervs og Uddannelses Foundation and Spar Nord Foundation for a grant covering the data collection and biomarker analysis. The foundation had  no influence on the study design, data interpretation, or manuscript writing.

## Data Availability

Data that support the findings of this study are available for the corresponding author upon request.
